# Clinical diagnosis and treatment of pediatric-onset relapsing polychondritis with airway involvement

**DOI:** 10.3389/fped.2025.1548142

**Published:** 2025-05-13

**Authors:** Ying Wang, Zhibo Xie, Jiarui Chen, Xiaoyan Li

**Affiliations:** Department of Otorhinolaryngology Head and Neck Surgery, Shanghai Children’s Hospital, School of Medicine, Shanghai Jiao Tong University, Shanghai, China

**Keywords:** relapsing polychondritis, laryngeal and tracheal stenosis, bioabsorbable corticosteroid-eluting stent implantation, laryngotracheal reconstruction, pediatric

## Abstract

**Objective:**

We herein retrospectively analyzed the clinical characteristics and treatment protocols of children with relapsing polychondritis (RP) with airway involvement.

**Methods:**

We reviewed the medical records of eight children with pediatric-onset RP with airway involvement who presented to Shanghai Children's Hospital from June 2021 to June 2024. All children met Damiani's criteria for the diagnosis of RP. One child underwent “laryngotracheal reconstruction with hyoid graft + T-tube implantation + bioabsorbable corticosteroid-eluting stent implantation,” while five children underwent “balloon dilatation + T-tube implantation + bioabsorbable corticosteroid-eluting stent implantation.” After the initial surgery, follow-up was performed every two months for a total of six months. Three bioabsorbable corticosteroid-eluting stents were placed in the upper left and upper and lower right T-tubes during this time.

**Results:**

All eight children were seen in our department for outpatient follow-up: one child was extubated; five children remained in outpatient follow-up; and the remaining two children continued to be treated in the rheumatology and immunology department due to poor control of their primary disease.

**Conclusion:**

Tracheotomy can be used to rapidly improve symptoms of dyspnea in children with RP disease progression. In the stable stage of the disease, the minimally invasive surgical method of “balloon dilatation + T-tube implantation + bioabsorbable corticosteroid-eluting stent implantation” was adopted to reduce secondary injury caused by surgical trauma (to the extent possible) and to improve the survival and quality of life of the children.

## Introduction

Relapsing polychondritis (RP) is a rare autoimmune disease first described by Jaksch Wartenhorst in 1923 (1). It is characterized by recurrent and degenerative inflammation at affected sites that can involve cartilage and connective tissues of the auricles, inner ears, nose, throat, trachea, bronchus, eyes, joints, and cardiovascular tissues (2). Recurrent polychondritis tends to occur in middle-aged individuals between 40 and 55 years of age (3) and rarely occurs in children—with most cases reported as individual cases (4). The incidence of recurrent polychondritis with airway involvement is more common in children than in adults, and the tracheotomy rate is high—reaching 1 in 3—with a lack of specific clinical indications and a poor prognosis (5). Laryngotracheal reconstruction is usually indicated for patients with limited stenosis of the trachea or subglottis, especially in adults. However, for relapsing polychondritis-induced laryngeal and tracheal stenosis, this reconstruction constitutes a novel treatment that has not yet been reported.

In this paper, we retrospectively analyzed the clinical characteristics and protocol for treating children with RP and airway involvement in our department. We recommend that early diagnosis and personalized treatment measures are of great significance for airway management and improving the quality of life of these children.

## Material and methods

We reviewed the medical records of eight children with airway involvement of RP who presented to our hospital between June 2021 and June 2024. Children demographics (sex, age), principal clinical features, time of diagnosis, diagnostic criteria, Myer–Cotton degree (6), enhanced computerized tomography (CT), and treatment and clinical follow-up were recorded (Table 1). This study was approved by the Institutional Review Board of Shanghai Children's Hospital (2021R053-E01), and written informed consent was obtained from the parents or guardians of all patients.

**Table 1:** Characteristics of patients with relapsing polychondritis with airway involvement.

Patients									
Clinical features	1	2	3	4	5	6	7	8	Mean (±SD)/frequency (%)
Sex	M	M	F	M	M	M	F	F	5/3 (SR)
Age, years	15	15	8	17	11	18	17	14	14.38 ± 3.38
Diagnostic delay, months	36	6	10	3	3	3	3	2	8.25 ± 11.51
Auricular chondritis	+	+	−	+	+	+	+	+	87.5%
Nasal chondritis	−	−	−	−	+	+	+	−	37.5%
Laryngeal chondritis	+	+	+	+	+	+	+	+	100%
Other sites of chondritis	−	−	−	−	−	−	−	−	0%
Joint pain/arthritis	+	−	-	−	−	−	−	−	12.5%
Ocular inflammation	−	−	−	−	−	−	−	−	0%
Cardiac involvement	−	−	−	−	−	−	−	−	0%
Laboratory test									
ANA	−	−	+	−	+	−	−	+	37.5%
RF	−	−	+	+	+	−	−	−	37.5%
Biopsy	−	−	+	+	−	+	−	+	50%
Evolution									
Tracheal collapse	+	+	+	+	+	+	+	+	100%
Myer–Cotton	IV	IV	III	IV	IV	IV	IV	III	/
Tracheostomy	+	+	+	+	+	+	+	+	100%
Treatment									
Corticosteroids	+	+	+	+	+	+	+	+	100%
Cyclophosphamide	+	+	−	+	+	+	+	+	87.5%
Biologics	+	−	−	−	−	+	+	+	50%
Surgery	L	B	B	B	/	B	B	/	/
Prognosis	Follow- up	Extubation	Follow- up	Follow- up	Follow- up	Follow- up	Follow- up	Follow- up	/

RF, rheumatoid factor; ANA, antinuclear antibodies; SR, sex ratio; L, laryngotracheal reconstruction with hyoid graft + T-tube implantation + bioabsorbable corticosteroid-eluting stent implantation; B, balloon dilatation + T-tube implantation + bioabsorbable corticosteroid-eluting stent implantation.

According to Damiani's criteria (7), the clinical features were as follows: (1) recurrent chondritis of both auricles; (2) non-erosive inflammatory arthritis; (3) chondritis of the nasal cartilages; (4) ocular inflammation that included conjunctivitis, scleritis, episcleritis, and/or uveitis; (5) chondritis of the respiratory tract that involved the laryngeal and/or tracheal cartilages; and (6) cochlear and/or vestibular damage manifested by sensorineural hearing loss, tinnitus, and/or vertigo. The presence of three or more of the aforementioned clinical features and the presence of one clinical feature with pathologic confirmation of the lesion site or lesions involving two or more anatomical sites responsive to glucocorticoid therapy were clinically diagnosed as relapsing polychondritis.

There were two surgical options. One of the operations was “laryngotracheal reconstruction with hyoid graft + T-tube implantation + bioabsorbable corticosteroid-eluting stent implantation.” The surgical procedure was as follows: A transverse incision was made at the original tracheotomy wound, and 2–4 tracheal rings, cricoid cartilage, thyroid cartilage, and hyoid bone were completely exposed. A portion of the narrow cricoid cartilage segment was completely removed by electrocoagulation using an electrotome, and the defective segment of the cricoid cartilage was repaired using the middle hyoid bone, which was trimmed. A T-tube (Boston Medical Products, Inc.) was inserted to prevent stenosis, and three drug-loaded stents (Puyi Shanghai Biotechnology Co., Ltd.) were placed around the tube. The second operation was “balloon dilatation + T-tube implantation + bioabsorbable corticosteroid-eluting stent implantation.” The surgical procedure was as follows: A balloon of appropriate size (balloon dilatation tube, Jiangsu Wedekang Medical Technology Co., Ltd.) was placed at the stenosis for dilation based on endoscopic exploration under the glottis. An applied pressure of 8 mPa was repeated three times for 30 s each; a T-tube (Boston Medical Products, Inc.) was inserted to prevent stenosis, and three drug-loaded stents (Puyi Shanghai Biotechnology Co., Ltd.) were placed around the T-tube.

## Results

Eight children with relapsing polychondritis with airway involvement were admitted to our department (Table 1), comprising five boys and three girls aged 8–17 years, with an average age of 13.2 years. The shortest time from the first appearance of symptoms to diagnosis was two months, and the longest was 36 months, with an average of 8.25 months. One child underwent “laryngotracheal reconstruction with hyoid graft + T-tube implantation + bioabsorbable corticosteroid-eluting stent implantation,” while five children underwent “balloon dilatation + T-tube implantation + bioabsorbable corticosteroid-eluting stent implantation.” All five children recovered well after surgery and were discharged successfully. All children underwent “laryngotracheal exploration + endotracheal drug stent implantation” every two months in the hospital after discharge. After repeating this treatment for two cycles, the T-tube was removed and replaced with a tracheal cannula, and tube plugging was attempted. All eight children experienced outpatient follow-up in our department: one child was extubated, five cases are still in outpatient follow-up, and the remaining two cases are still undergoing systemic treatment by Rheumatology and Immunology specialists due to poor control of their primary disease, and surgeries have been scheduled.

### Patient medical reports

**Figure 1:**
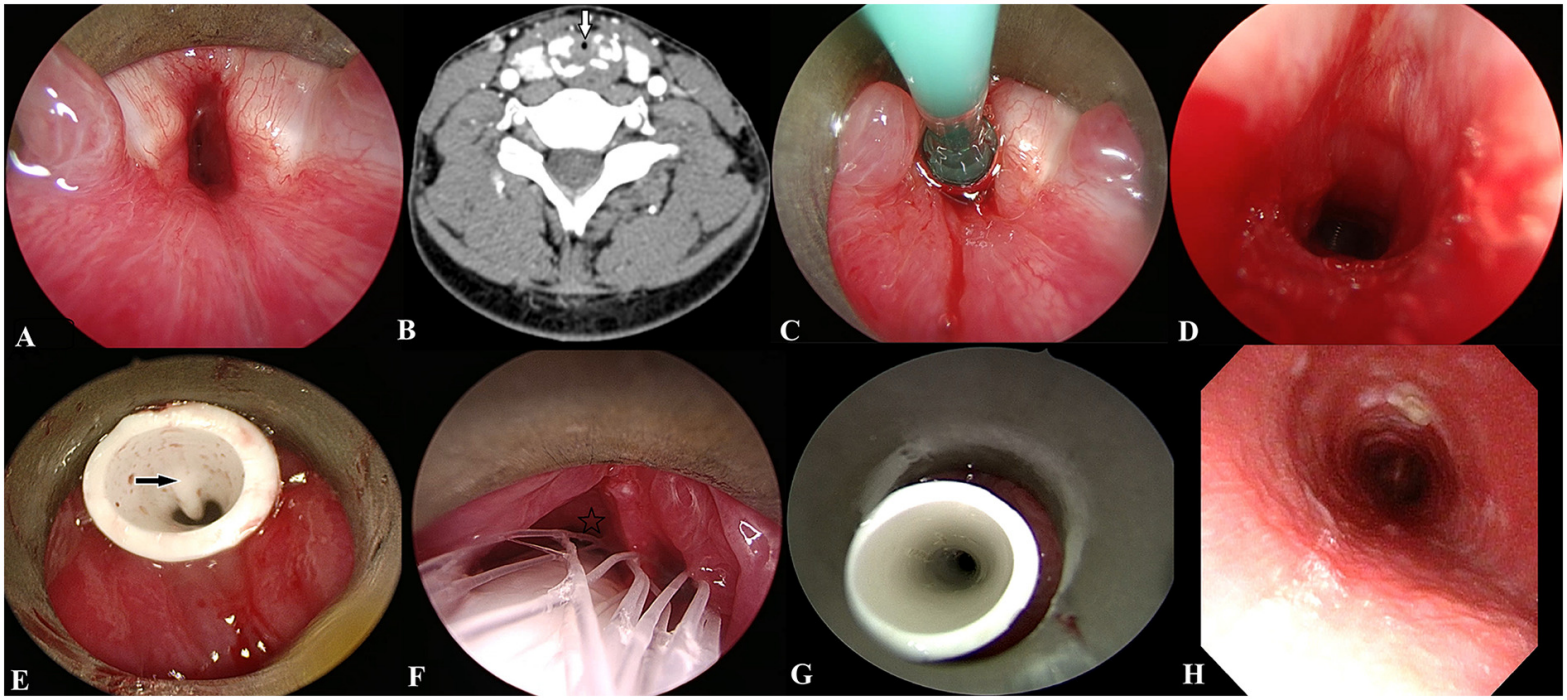
Case 2 before and after treatment. **(A)** Preoperative endoscopy: subglottic mucosal swelling, airway stenosis. **(B)** Head and neck CT showed a thickening of the tracheal wall (white arrow) and laryngotracheal cartilage collapse, calcification, and deformity. **(C)** A balloon of appropriate size was placed at the stenosis for dilation according to the endoscopic exploration under the glottis. **(D)** Subglottic stenosis improved rapidly after balloon dilation. **(E)** After T-tube implantation. The black arrow indicates the inner wall of the T-tube fold. **(F)**. Two months after the operation. A drug-loaded stent was implanted between the T-tube and the mucosa of the airway wall, and the asterisk indicates the mucosal gap between the T-tube and the airway wall. **(G)** Six months after the operation. The inner wall of the T-tube fold disappeared. **(H)** Subglottic airway was smooth after extubation.

A 15-year-old male was treated at our hospital for “sudden dyspnea with laryngeal wheezing” and was given assisted ventilation with a tracheal intubation ventilator. When repeated attempts at extubation failed, a tracheotomy was performed, and an endoscopy revealed swelling of both vocal cords and subglottic stenosis. The boy was subsequently diagnosed with RP by Damiani's criteria, and pathologic examination of an auricle biopsy revealed marked infiltration of neutrophils in and around the cartilage, with degeneration. After treatment with intravenous methylprednisolone pulse therapy followed by oral prednisolone and immunosuppressants (methotrexate) in our Rheumatology Department, his general condition stabilized, and he was admitted to our department because of tracheal cannula extubation failure. Endoscopy showed swelling and thickening of the mucosa under the glottis, and the subglottic stenosis showed IV° according to the Myer–Cotton degree (Figure 1A). A bronchoscopic examination showed smoothness at the lower portion of the trachea, but a slight stenosis of the lumen of each primary bronchus. Head and neck CT revealed thickening of the tracheal wall and laryngotracheal cartilage collapse, calcification, and deformity. “Balloon dilatation + T-tube implantation + bioabsorbable corticosteroid-eluting stent implantation” was performed and a T-tube was inserted to maintain the tracheal lumen. A folding of the inner wall of the T-tube was observed due to the mucosal swelling of the surrounding airway wall (Figure 1E). The boy was seen by his physician every two months after surgery, and the physician noted that the inner wall folds of the T-tube disappeared and that the gap between the T-tube and the surrounding trachea wall increased significantly (Figure 1G). After repeated treatments for three cycles, the T-tube was replaced with a tracheal cannula. After three months with the tracheal cannula capped, a re-examination endoscopy indicated that the tracheal was smooth above the cannula (Figure 1H), and the boy was extubated and discharged successfully.

## Discussion

The incidence of relapsing polychondritis ranges from 0.71 to 9 per million, with no significant sex or racial differences (8). The disease can occur in children of all ages (1.7 months to 17 years), and the misdiagnosis rate is high, with a median time from first symptom to diagnosis ranging from 1.9 to 3.2 years (2, 9). Systemic symptoms include fever, fatigue, and weight loss, while local symptoms vary depending on the cartilage involved. Patients with RP of airway involvement is commonly attributed to inflammation and leads to airway narrowing and/or the loss of cartilaginous structural support, particularly in the subglottic region and trachea (4). Repeated collapse of airway cartilage and pulmonary infection are the major factors leading to death in patients with RP. Dysphonia, hoarseness of the voice, or inspiratory dyspnea is also possible when the laryngeal cartilage is involved. Tracheobronchial involvement is manifested by progressive dyspnea, cough, stridor, and even respiratory distress (10). According to reports in the literature, approximately 50% of patients with relapsing polychondritis show differing degrees of airway involvement during the progression of the disease (11). The authors of a retrospective analysis of 142 patients with RP in France (12) found that 43% harbored laryngeal cartilage involvement and 22% had tracheal and bronchial cartilage involvement. There were also large studies in China (13, 14) that involved a total of 505 patients with relapsing polychondritis, and their results revealed that 69–81.7% of their patients possessed airway involvement to varying degrees.

The pathologic manifestations of relapsing polychondritis with airway involvement differ at various stages of disease progression. Airway inﬂammatory swelling occurs in the active stage, followed by malacia that results from cartilage destruction and stenosis due to ﬁbrous replacement of the impaired cartilage (10, 15). The tracheal or bronchial wall is thickened to greater than 2 mm with or without calcification on CT, and there is fixed lumen narrowing and/or tracheobronchial obstruction that includes subglottic stenosis. The obstructive diameter of the affected airway lumen can thus be reduced by at least 25% (16). The combination of endoscopy and CT scanning garners a good correlation in the diagnosis and differential diagnosis of tracheobronchial stenosis. However, in some children with atypical clinical symptoms—especially in children with RP with only airway involvement—local cartilage biopsy is difficult to procure. Positron emission tomography (PET)-CT has in recent years become an area of intense research focus in the diagnosis of RP. Investigators have found that RP manifests high metabolic activity in the cartilage of the affected site in 18F-fluorodeoxyglucose (FDG)-PET/CT images, and therefore postulated that the modality can be key to guiding biopsy, and its diagnostic accuracy can reach 93% (17).

Large doses of glucocorticoids can be used to quickly control airway swelling and collapse caused by acute mucosal edema to alleviate the clinical symptoms of dyspnea, and immunosuppressants may be added when glucocorticoids alone do not provide adequate relief. With systemic stability, active surgical intervention is of paramount importance for airway management and the improvement of patient's quality of life. We herein ascertained that respiratory symptoms were commonly attributed to inflammation, leading to airway narrowing and/or the loss of cartilaginous structural support—particularly in the subglottic region and trachea. Therefore, the determination of optimal timing and method of surgical intervention is very important. In the progression of the disease, tracheotomy can rapidly improve the symptoms of dyspnea in children in a short period of time and provide opportunities for follow-up systemic drug therapy. When the systemic symptoms are stable, surgical intervention is selected. The stable phase of the disease includes discontinuation of systemic immunosuppressants and glucocorticoids or maintenance of the lowest dose of glucocorticoids while systemic symptoms are controlled. Balloon dilatation with adjustable pressure and security performance is suitable for relapsing polychondritis-induced laryngeal and tracheal stenosis.

“Laryngotracheal reconstruction with hyoid graft + T-tube implantation + bioabsorbable corticosteroid-eluting stent implantation” is not recommended for laryngotracheoplasty. In contrast to adults, children have poor tolerance to surgery. Long-term and traumatic surgery tends to aggravate the systemic symptoms of children with RP to a degree, and this is not conducive to the control of the primary disease. One child who underwent this type of surgery (case 1) had a recurrence of auricle perichondritis, and the systemic symptoms of the primary disease tended to worsen; we therefore recommend “balloon dilatation + T-tube implantation + bioabsorbable corticosteroid-eluting stent implantation” for this type of child. The procedure's advantages are as follows: (1) The inner diameter of the subglottic region and trachea was temporarily enlarged by balloon dilation, and the T-tube implantation was integral in supporting the collapsed cartilage. (2) The placement time of the T-tube was six months, and its support elicited a continuously expansive effect on the mucosa and cartilage at the stenosis of the airway. (3) The support and fixation of the T-tube lessen the risk of the surrounding drug stent shifting or falling off. (4) The drug scaffold is a biodegradable biopolymer coated with glucocorticoids, and during the degradation process, the drug is released slowly and quantifiably, thereby reducing postoperative inflammation and scarring. The degradation time is approximately two months, with a portion of the scaffold absorbed by the tissue and the rest entering the digestive system with swallowing and discharge from the body. Our protocol differs from that with expandable metallic stents as it avoids granulomas, obstructive granulomas, stent migration, fracture, and mucus plugging (15, 18). (5) We performed drug stent implantation again at the two-month follow-up after surgery to reduce the dosage of systemic glucocorticoids via the sustained slow-release effect of the local glucocorticoids on airway inflammation thereby alleviating adverse reactions in the children. When a patient was implanted with the T-tube in the first operation, the T-tube could not fully expand, and the lumen was compressed and distorted due to severe swelling of the tracheal mucosa and narrowing of the lumen. With the slow-release action of the surrounding drug stent, the swelling of the airway mucosa was significantly improved, the T-tube lumen was completely stretched, and the gap between the T-tube and the mucosa around the trachea was enlarged, showing remarkable results (Figure 1).

Relapsing polychondritis is a rare autoimmune disease with a low incidence. Only eight children were included in this study, and the small number of cases restricts its wide application. At present, only one boy has been successfully extubated, and thus it is necessary to extend the follow-up period and evaluate long-term effects. However, despite this, this surgical approach is indeed a novel option for children with RP with airway involvement due to its low trauma and rapid postoperative recovery.

## Conclusion

In pediatric-onset relapsing polychondritis with airway involvement, surgical intervention is more difficult, and there is a paucity of unified treatment standards; thus, the choice of operative timing and operational mode is particularly important. Tracheotomy can be used in the progression of the disease to rapidly improve the symptoms of dyspnea in children. In the stable stage of the disease, the minimally invasive surgical method of “balloon dilatation + T-tube implantation + bioabsorbable corticosteroid-eluting stent implantation” was adopted to reduce as much as possible secondary injury caused by surgical trauma and thereby improve the survival and quality of life of the children.

## Data Availability

The raw data supporting the conclusions of this article will be made available by the authors, without undue reservation.
